# The time course of stimulus-specific perceptual learning

**DOI:** 10.1167/jov.24.4.9

**Published:** 2024-04-11

**Authors:** Patrick J. Bennett, Ali Hashemi, Jordan W. Lass, Allison B. Sekuler, Zahra Hussain

**Affiliations:** 1 Department of Psychology, Neuroscience, and Behaviour, McMaster University, Hamilton, Canada; 2 Rotman Research Institute, Baycrest Centre for Geriatric Care, Toronto, Canada; 3 Department of Psychology, University of Toronto, Toronto, Canada; 4 School of Psychology, University of Plymouth, Plymouth, UK

**Keywords:** vision, perceptual learning, pattern identification, psychophysics, individual differences

## Abstract

Practice on perceptual tasks can lead to long-lasting, stimulus-specific improvements. Rapid stimulus-specific learning, assessed 24 hours after practice, has been found with just 105 practice trials in a face identification task. However, a much longer time course for stimulus-specific learning has been found in other tasks. Here, we examined 1) whether rapid stimulus-specific learning occurs for unfamiliar, non-face stimuli in a texture identification task; 2) the effects of varying practice across a range from just 21 trials up to 840 trials; and 3) if rapid, stimulus-specific learning persists over a 1-week, as well as a 1-day, interval. Observers performed a texture identification task in two sessions separated by one day (Experiment 1) or 1 week (Experiment 2). Observers received varying amounts of practice (21, 63, 105, or 840 training trials) in session 1 and completed 840 trials in session 2. In session 2, one-half of the observers in each group performed the task with the same textures as in session 1, and one-half switched to novel textures (same vs. novel conditions). In both experiments we found that stimulus-specific learning – defined as the difference in response accuracy in the same and novel conditions – increased as a linear function of the log number of session 1 training trials and was statistically significant after approximately 100 training trials. The effects of stimulus novelty did not differ across experiments. These results support the idea that stimulus-specific learning in our task arises gradually and continuously through practice, perhaps concurrently with general learning.

## Introduction

Perceptual learning refers to the improvement in performance in perceptual tasks that occurs as a result of practice (Fiorentini & Berardi, 1981; Ball & Sekuler, 1987; Karni & Sagi, 1991; Ahissar & Hochstein, 1996; Hussain, Sekuler, & Bennett, 2009b). It often is long-lasting (Ball & Sekuler, 1982; Karni & Sagi, 1993; Adini, Sagi, & Tsodyks, 2002; Hussain, Sekuler, & Bennett, 2011; Yashar, Chen, & Carrasco, 2015) and exhibits some degree of specificity for the stimuli used during training (Dresslar, 1894; Zwislocki, Maire, Feldman, & Rubin, 1958; Ball & Sekuler, 1982; Karni & Sagi, 1991; Poggio, Fahle, & Edelman, 1992; Ahissar & Hochstein, 1996; Hussain, Sekuler, & Bennett, 2009a; Jeter, Dosher, Liu, & Lu, 2010; Hussain et al., 2011). In many tasks, perceptual performance improves rapidly during the first several trials and then improves more slowly over hundreds and sometimes thousands of trials (Doane & Alderton, 1996; Karni & Bertini, 1997; Ortiz & Wright, 2009; Hussain et al., 2009b). Some have suggested that the rapid initial changes in performance are due to participants learning about the task (Karni & Bertini, 1997; Karni et al., 1998; Saffell & Matthews, 2003; Wright & Fitzgerald, 2001; Ortiz & Wright, 2009), whereas the slower, subsequent changes in performance reflect stimulus-specific learning (Ramachandran & Braddick, 1973; Fiorentini & Berardi, 1981; Ball & Sekuler, 1987; Karni & Sagi, 1991; Poggio et al., 1992; Fahle, Edelman, & Poggio, 1995; Ahissar & Hochstein, 1996). Ahissar & Hochstein (1997), for example, proposed that general learning emerges first and is used as a guide for later stimulus-specific learning.

Nevertheless, performance in a wide variety of tasks is approximately a power or exponential function of practice (Heathcote, Brown, & Mewhort, 2000). In such cases, response accuracy is approximately proportional to the logarithm of the number of practice trials and there are no obvious discontinuities in the learning curve that might indicate fast and slow phases of learning (Dosher & Lu, 2005; Dosher & Lu, 2007; Hussain et al., 2009b). Such findings suggest that stimulus-specific learning may begin right at the start of practice, perhaps concurrently with general learning about the task. One way of testing this idea is to vary the amount of practice before participants switch to novel stimuli. If stimulus-specific learning begins at the start of training, then we would expect the effect of stimulus novelty (i.e., the difference in performance between groups who see the same versus novel stimuli) in the test session to increase as a monotonic function of the number of training trials, and studies with sufficient statistical power would find a statistically significant effect of novelty in groups that receive relatively few training trials.

The results of studies using this method have been mixed. Jeter et al. (2010) found that learning in an orientation discrimination task generalized to novel stimuli early on in practice (after approximately 1,200 trials performed in one day) and that stimulus specificity emerged only after extensive training (over 7,000 trials performed over several days), with less specificity after intermediate amounts of practice. On the other hand, Hussain, McGraw, Sekuler, & Bennett (2012) found that 105 and 840 practice trials in a 1-of-10 face identification task produced equivalent amounts of stimulus-specific learning 24 hours after the initial training. Although the Hussain et al. (2012) result might reflect specialized learning mechanisms for faces, similarly rapid stimulus-specific learning has been found for discrimination of auditory stimuli in some studies (Hawkey, Amitay, & Moore, 2004; Wright, Wilson, & Sabin, 2010). Indeed, Wright et al. (2010) found more stimulus-specific learning with small than large amounts of practice, contrary to the work cited earlier. Finally, Aberg, Tartaglia, & Herzog (2009) found that stimulus-specific learning in a visual chevron discrimination task depended on how practice trials were distributed across sessions. Specifically, stimulus specificity was found with 1,600 practice trials divided equally into two sessions separated by 1 day, but stimulus generalization was found with 1,600 trials divided equally into four sessions separated by 1 week. Hence, the time course of stimulus-specific learning seems to depend on the perceptual task and perhaps on the interval between training and test sessions.

Given the results suggesting that specificity of learning depends both on the amount of practice and the temporal interval between sessions, and to test whether the early specificity found for faces is found for identification tasks more generally, we decided to conduct the same experiment as Hussain et al. (2012) with two variations. We used the same task (1-of-10 identification), but with non-facial stimuli, namely, random textures, which are more in line with stimuli used in other studies. Additionally, we evaluated learning with two different intervals between sessions: 1 day (Experiment 1), and 1 week (Experiment 2). Finally, we sought to provide a more detailed analysis of the time course of rapid learning by measuring the effects of 21, 63, 105, and 840 practice trials (i.e., two more conditions than were tested by Hussain et al., 2012).

## Experiment 1 (1-day interval)

### Methods

#### Participants

Ninety-six naïve undergraduate students from McMaster University (21 males, mean age = 19.7 years, range = 18–30 years) participated in the experiment. All participants had normal or corrected-to-normal visual acuity, and were either paid $10/hour or given partial course credit for participating. The experimental protocol for the experiment was approved by the McMaster University Research Ethics board, and informed consent was obtained from each participant prior to the experiment.

#### Apparatus and stimuli

Stimuli were generated on an Apple Macintosh G4 computer using MATLAB and the Psychophysics and Video toolboxes (Brainard, 1997; Pelli, 1997). Stimuli were presented on a 19-inch NEC MultiSync FE992 display with a resolution of 1,280 × 1,024 pixels (36 pixels/cm), and a refresh rate of 85 Hz. Participants viewed the display binocularly from a distance of 88 cm, and a chin/forehead rest was used to stabilize viewing position. The display had an average luminance of 82 cd/m^2^ and was the only source of illumination in the testing room.

**Figure 1.**
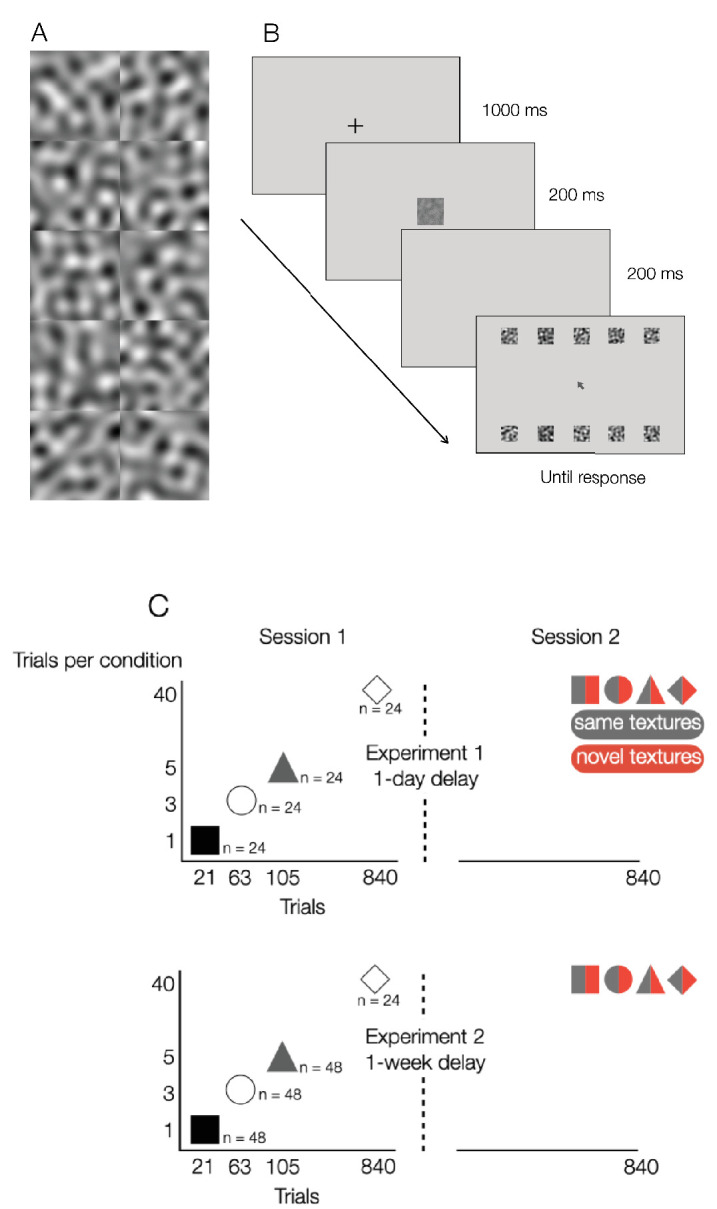
(A) The stimuli were two sets of 10 textures (only one set is shown) that were constructed by filtering white noise with an isotropic, band-pass spatial frequency filter. Across trials, textures were presented at seven different contrasts in three levels of external noise. (B) Each trial consisted of a central fixation point followed by a single, target texture embedded in noise and a response screen consisting of noiseless, high-contrast thumbnail images of the 10 possible textures. The target was selected randomly on each trial. Observers identified the target by clicking on a thumbnail image with a computer mouse. (C) The experiment consisted of two sessions separated by 1 day (Experiment 1) or 1 week (Experiment 2). In session 1, participants performed the identification task for 21, 63, 105, or 840 trials (or 1, 3, 5, or 40 21-trial blocks). In session 2, all participants completed 840 trials: one-half saw the same textures that were presented in session 1, and one-half saw a novel set of 10 textures. The symbols representing session 1 training correspond with the symbols used in Figures 6 and 7.

The textures were 2 sets of 10 band-limited noise patterns created by applying an isotropic, band-pass (2-4 cy/image) ideal spatial frequency filter to a 256 × 256 pixel (4.6° × 4.6°) patch of Gaussian noise (Figure 1A). Each texture was presented at seven contrast levels that were equally spaced on a logarithmic scale. The stimuli were embedded in three levels of static two-dimensional Gaussian noise with contrast variances of 0.001, 0.01, and 0.1. The 7 stimulus contrasts and 3 levels of noise yielded a total of 21 stimulus conditions which spanned the sub-threshold to supra-threshold range.

#### Procedure

Each participant began the experiment with a 60-second period during which they adapted to the luminance of the display. The sequence of events in a trial is illustrated in Figure 1B. Each trial began with a small, high-contrast fixation point presented in the middle of the screen for 1 second, followed by the presentation of a target texture stimulus for 200 ms. After a 200 ms blank screen, a response screen consisting of thumbnail versions of the 10 possible textures (each 2.3° × 2.3°) appeared in a 2 × 5 array. On each trial, the target was selected randomly from the set of 10 possible textures, and the participant’s task was to identify the target stimulus by clicking on 1 item in the response screen with a computer mouse. The response screen remained visible until a response was made, auditory feedback was provided for correct (800-Hz tone) and incorrect (200-Hz tone) responses, and the next trial started immediately following the auditory feedback. Trial order was randomized, with the constraint that each combination of Gaussian noise variance and stimulus contrast was presented only once in each block of 21 trials. Across trials, the 10 textures were always shown in the same locations in the response screen. The average luminance of the display remained constant throughout the testing procedure.

#### Design

The experimental design is illustrated in Figure 1C. Each participant was tested in two sessions separated by one day. In session 1, participants each completed 1, 3, 5, or 40 21-trial blocks (i.e., 21, 63, 105, or 840 trials, respectively). Twenty-four participants were assigned to each group. In session 2, all participants completed 40 blocks, or a total of 840 trials. There was no delay between blocks (i.e., the session progressed as a continuous run of trials). In addition, in session 2 one-half of the participants were presented with the same set of textures seen in session 1 and the other one-half saw a novel set of textures. In the remainder of this paper, we refer to these groups as same-21 and novel-21, same-63 and novel-63, and so on. Note that participants were not given any preliminary practice on the task in either session 1 or 2, and that no trials in either session were discarded as preparatory trials.

### Results

All statistical analyses were performed with R (R Core Team, 2021). Effect size (Cohen’s *d*, *f*, and partial *f*) was calculated using R’s *effectsize* package (Ben-Shachar, Lüdecke, & Makowski, 2020), and effects plots (e.g., Figures 4, 6, and 7) were generated with the *effects* package (Fox, 2003). Linear mixed-effects models were fit to data with the *lme4* and *lmerTest* packages (Bates, Mächler, Bolker, & Walker, 2015; Kuznetsova, Brockhoff, & Christensen, 2017). Analyses on arcsine-transformed and untransformed response accuracy (i.e., proportion correct) yielded similar results and therefore we present the analyses of untransformed data.

**Figure 2.**
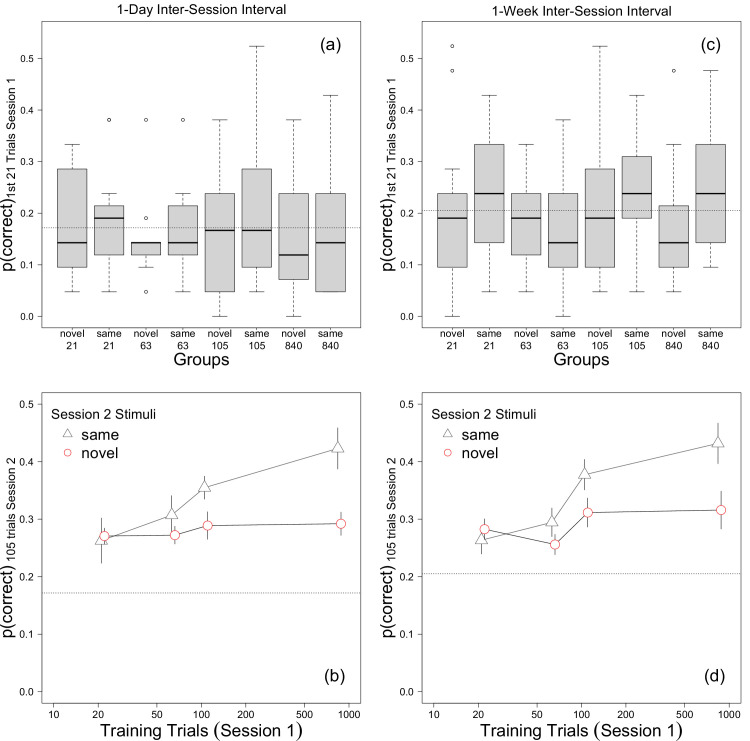
Response accuracy from Experiments 1 (left) and 2 (right). (Top) The proportion of correct responses in the first 21 trials in session 1. (Bottom) Proportion correct in the first 105 trials in session 2 plotted against session 1 training trials and session 2 stimulus novelty. Error bars in b and d represent ±1 SEM. The horizontal dashed line in each plot indicates the average proportion correct in the first 21 trials in session 1.

**Figure 3.**
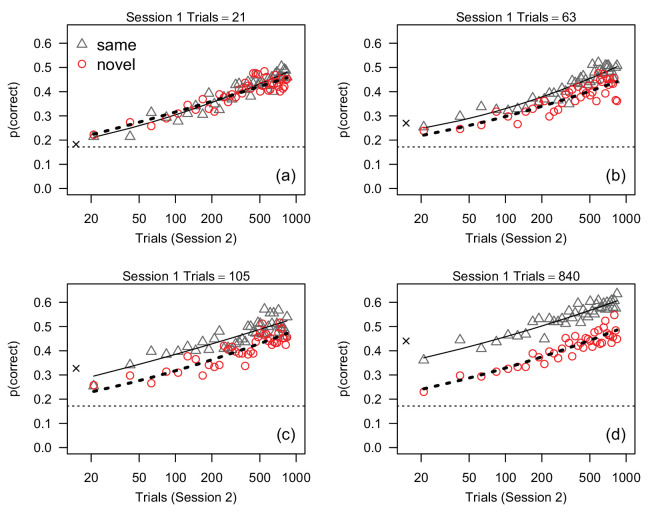
Proportion correct, averaged across participants, measured in 40 21-trial bins in session 2 in Experiment 1 (1-day interval). Each plot shows data from participants who received different amounts of training in session 1. Solid and dashed lines represent the predictions of the best-fitting linear mixed-effects model (Equation 1) for the same and novel conditions, respectively. The dotted horizontal line in each plot indicates the average proportion correct during the first 21 trials in session 1. The × symbol on the far left of each plot shows average accuracy in the last 21 trials in session 1.

**Figure 4.**
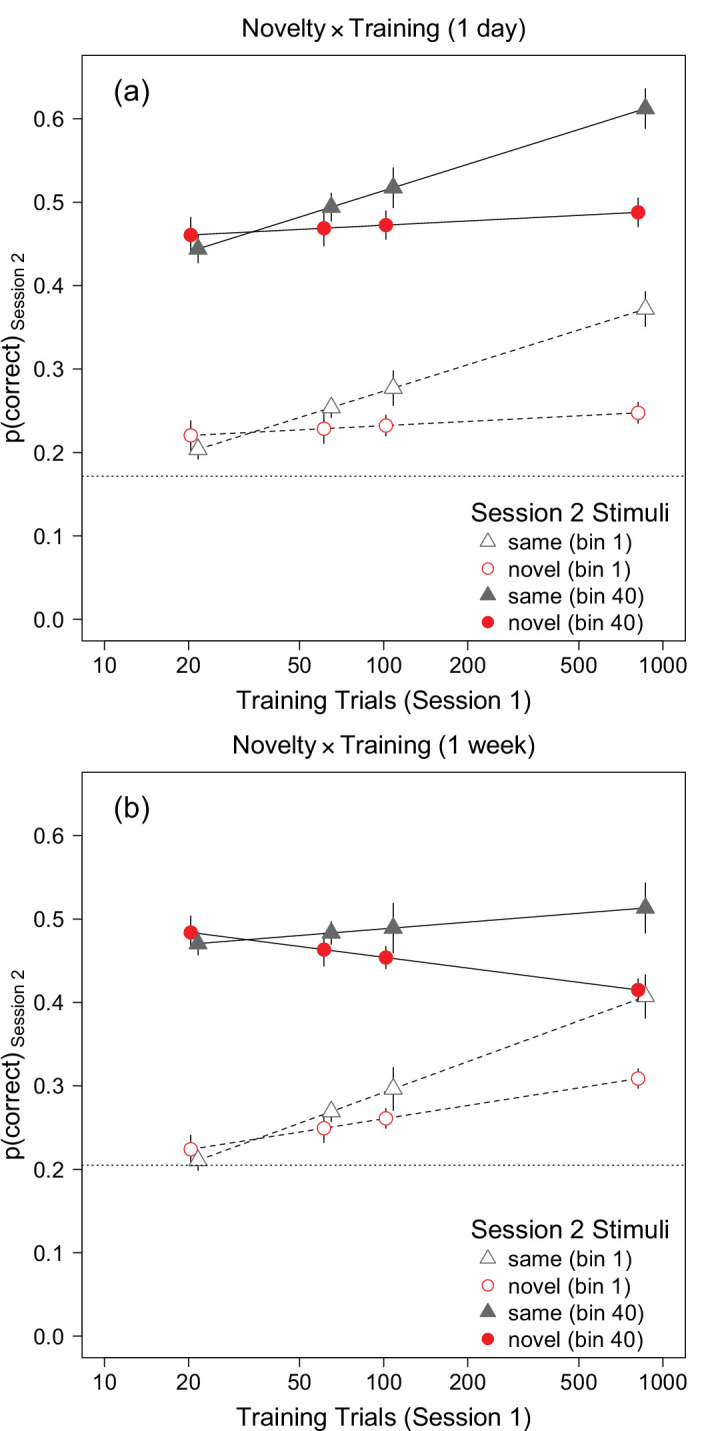
Effect plots illustrating the novelty × training interaction found by the mixed-effects model analysis of Experiment 1 (a) and Experiment 2 (b). Each point is estimated accuracy in session 2 at bin 1 (*t*_2_ = 21) or bin 40 (*t*_2_ = 840) after setting $p_{1}$ to its mean. In both panels, the difference between the same and novel conditions is proportional to log (*T*_1_). The horizontal dotted line is average proportion correct during the first 21 trials in session 1. Error bars represent ±1 SEM. The points have been shifted horizontally slightly for clarity.

**Figure 5.**
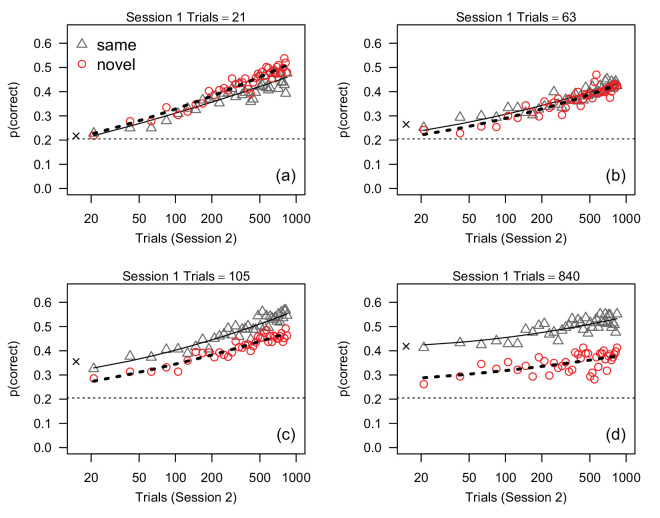
Response accuracy from Experiment 2 (1-week interval), averaged across participants, measured in 40 21-trial bins in session 2. Each plot shows data from participants who received different amounts of training in session 1. The solid and dashed lines represent the predictions of the best-fitting linear mixed-effects model (Equation 2) for the same and novel conditions, respectively. The horizontal dashed line in each plot indicates the average proportion correct during the first 21 trials in session 1. The × symbol on the far left of each plot shows average accuracy in the last 21 trials session 1.

**Figure 6.**
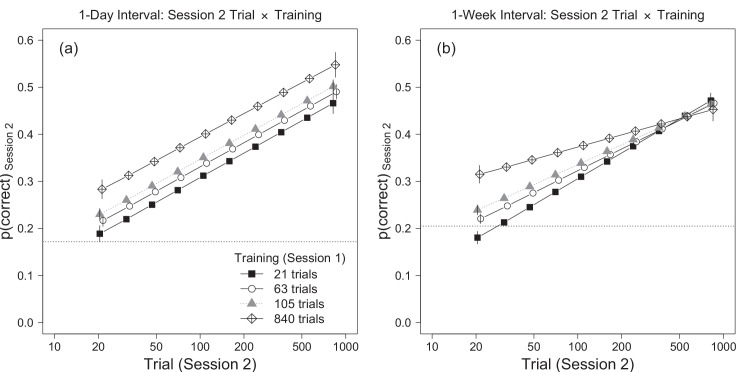
Illustration of the trial × training × experiment interaction from the mixed-effects model analysis of the combined data from Experiments 1 and 2 (Table A4). Each point is estimated accuracy in session 2, averaged across novelty conditions, with the covariate ($p_{1}$) set to its mean. The horizontal dotted lines indicate mean values of $p_{1}$. Error bars are ±1 SEM. (a) In Experiment 1 the effect of training persisted throughout session 2 and the trial × training interaction was not significant. (b) In Experiment 2 the effect of training diminished with increasing trial number and the trial × training interaction was significant.

**Figure 7.**
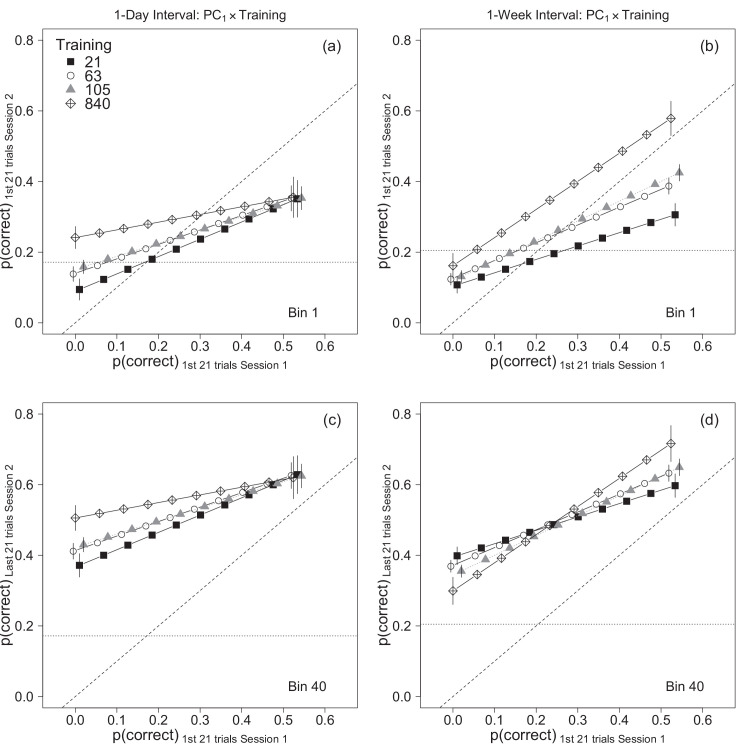
Illustration of the $p_{1}\times \mathrm{training}\times \mathrm{experiment}$ interaction from the mixed-effects model analysis of the combined data from Experiments 1 and 2 (Table A4). Each point is estimated accuracy, averaged across novelty conditions, in the first 21 trials (top) and last 21 trials (bottom) in session 2. Results for Experiment 1 and 2 are shown in the left and right columns, respectively. The horizontal dotted line in each plot indicates the mean value of $p_{1}$, and the diagonal line represents *y* = *x*. Error bars represent ±1 SEM. The various traces have been shifted horizontally slightly for clarity.

#### Session 1: Accuracy

We first determined if initial performance in session 1 differed across groups. The average proportion of correct responses during the first 21 trials of session 1, which ranged from 0.15 to 0.21, is plotted in Figure 2a. A one-way analysis of variance (ANOVA) found that the effect of group was not significant, *F*(7, 88) = 0.33, *p* = 0.94, *f* = 0.16. Analyzing the data with a 2 (novelty) × 4 (training) ANOVA also found no significant effects, *F* ⩽ 0.91, *p* ⩾ 0.34, *f*_*p*_ ⩽ 0.10. Hence, initial accuracy in session 1 did not differ significantly across groups.

#### Session 2: Accuracy, the first 105 trials

We next analyzed accuracy in session 2. To make our analyses comparable with those reported by Hussain et al. (2012, i.e., the data in Figure 2, bin 9, in that paper), we first focused on the proportion of correct responses in the first 105 trials in session 2.

Figure 2b plots response accuracy on the first 105 trials in session 2 against the number of session 1 training trials separately for groups that saw the same and novel stimuli in session 2 (henceforth same vs. novel conditions). The horizontal dashed line in Figure 2b indicates the average proportion correct in the first 21 trials in session 1 and can be thought of as a baseline: points lying above the line are indicative of learning, and stimulus-specific learning is indicated by accuracy in the same conditions being higher than accuracy in the novel conditions. Accuracy in all conditions was well above baseline performance, demonstrating that learning occurred in all conditions. Also, accuracy in the same conditions, but not the novel conditions, was a monotonically increasing function of session 1 training trials. Hence, the effect of stimulus novelty increased with increasing session 1 training.

A 2 (novelty) × 4 (training) ANOVA on accuracy in the first 105 trials on session 2 found significant main effects of novelty, *F*(1, 88) = 8.65, *p* = 0.004, *f*_*p*_ = 0.31, and training, *F*(3, 88) = 4.32, *p* = 0.007, *f*_*p*_ = 0.38, and a non-significant novelty × training interaction, *F*(3, 88) = 2.36, *p* = 0.077, *f*_*p*_ = 0.28. Planned linear contrasts found that the linear trend of accuracy across log-transformed training trials was significant, *F*(1, 88) = 12.34, *p* < 0.001, *f*_*p*_ = 0.37, and the linear trend differed significantly between novelty conditions, *F*(1, 88) = 6.99, *p* = 0.009, *f*_*p*_ = 0.28. The linear trend was significant in the same condition, *F*(1, 44) = 12.5, *p* < 0.001, *f* = 0.54, but not in the novel condition, *F*(1, 44) = 0.77, *p* = 0.39, *f* = 0.13. The nonlinear trends were not significant, *F*(2, 88) = 0.31, *p* = 0.74, *f*_*p*_ = 0.08, and did not differ significantly between novelty conditions, *F*(2, 88) = 0.04, *p* = 0.96, *f*_*p*_ = 0.03. These trend analyses suggest that average accuracy was approximately proportional to the log-transformed number of training trials in the same condition but not the novel condition, and therefore that the difference between accuracy in the same and novel conditions increased with training trials. The results of *t* tests comparing accuracy in the same and novel conditions in each training condition are consistent with this idea: the effect of novelty was significant in the 840 trials, *t*(17.4) = −3.18, *p* = 0.005, *d* = 8.33, and 105 trials, *t*(21.3) = −2.11, *p* = 0.046, *d* = 0.92, conditions, but not in the 63 trials, *t*(15.4) = −0.94, *p* = 0.36, *d* = 0.48, or 21 trials, *t*(13.7) = −0.19, *p* = 0.85, *d* = 0.10, conditions. Hence, consistent with Hussain et al. (2012), stimulus-specific learning was obtained with 105 practice trials. In addition, the trend analyses suggest that stimulus-specific learning may begin even earlier during practice.

Hussain et al. (2012) found that stimulus-specific learning in a face identification task was equivalent in participants who received 105 and 840 training trials. We examined whether this was true for our experiment by analyzing the data from the 105-trials and 840-trials conditions with a 2 (novelty) × 2 (training) ANOVA. Consistent with Hussain et al. (2012), the main effect of novelty was significant, *F*(1, 44) = 15.52, *p* < .001, *f* = 0.57, and the novelty × training interaction, *F*(1, 44) = 1.592, *p* = 0.21, *f* = 0.19, was not significant, indicating that stimulus-specific learning did not differ significantly with 105 and 840 training trials. Unlike Hussain et al. (2012) the main effect of training, *F*(1, 44) = 1.91, *p* = 0.17, *f* = 0.21, was not significant.

#### Session 2: Time course and individual differences

So far, our analyses suggest that session 2 accuracy increased with session 1 training trials in the same condition but not the novel condition, and that a statistically significant difference between the same and novel conditions emerged after approximately 105 training trials. However, those analyses ignored individual differences in visual identification, which presumably also affected performance in session 2. We therefore reanalyzed our data with a model that took into account initial session 1 performance (Figure 2a). Furthermore, to obtain a more detailed description of how performance in session 2 was affected by stimulus novelty, training in session 1, and within-session improvements in session 2, we divided the 840 session-2 trials into 40 bins each consisting of 21 trials.

Figure 3 plots the proportion of correct responses for each 21-trial bin in session 2 separately for each group. Proportion correct for bin 1 (i.e., the first 21 trials in session 2) is plotted at session 2 trial number 21, proportion correct for bin 2 is plotted at trial number 42, proportion correct for bin 3 is plotted at trial number 63, and so forth. In all groups, average response accuracy was approximately proportional to the logarithm of the number of trials in session 2. There was no evidence of stimulus-specific learning in groups that received 21 trials of practice in session 1 (i.e., there was no apparent difference in performance between the same and novel groups). With 63 trials of practice there was some suggestion of a small stimulus-specific component of learning that persisted throughout the testing session in session 2, and this stimulus-specific effect was larger in the groups that received 105 and 840 training trials. This pattern is also evident with respect to average accuracy in the last bin of session 1: accuracy in the first bin of session 2 was roughly equivalent to accuracy in the last bin of session 1 for the same groups regardless of session-1 training, but showed a successively larger drop against session 1 for the novel group with increasing amounts of training. Figure S1 (Supplementary material) shows this pattern in more detail.

To determine how performance in session 2 was influenced by stimulus novelty, the number of session 1 training trials, and initial session 1 accuracy, the data in Figure 3 were analyzed with a linear mixed-effects model (Bates et al., 2015; Kuznetsova et al., 2017). The fixed predictor variables consisted of a two-level factor representing stimulus novelty in session 2 and four numeric variables representing i) proportion correct on the first 21 trials of session 1; ii) log-transformed session 1 training trials; and iii) linear and quadratic terms for log-transformed session 2 trials. Each numeric variable was centered on its mean. Initially we compared the goodness-of-fit obtained by three models that included all two-, three-, and four-way interactions between all of the terms: pairwise comparisons of goodness-of-fit failed to find a significant difference between these models (*p* ⩾ 0.30 for all χ^2^ tests) and therefore we focused on the simpler, two-way interaction model. Next, we dropped eight two-way interactions that did not approach statistical significance, *p* > 0.10. The goodness-of-fit for the reduced and full two-way models did not differ significantly, χ^2^ = 9.047, df=8, *p* = 0.34, and therefore we present results obtained with the simpler fixed-effects model defined by the equation

(1) $$\begin{array}{ccc} y & \;= & \mu_{0}+p_{1}+N+T_{1}+t_{2}+t_{2}^{2}\\ & & +\;\left(p_{1}\times N\right)+\left(N\times T_{1}\right)\;\; \end{array}$$

where *y* is proportion correct in a 21-trial bin in session 2, μ_0_ is the intercept, $p_{1}$ is proportion correct on the first 21 trials in session 1, *N* is stimulus novelty in session 2, *T*_1_ is log-transformed session 1 training trials, and *t*_2_ is log-transformed session 2 trial number divided into 40, 21-trial bins (i.e., trial 21, 42, 63, ⋅⋅⋅, 840). Equation 1 defines the curves that were drawn through the data points in Figure 3. Finally, the height, slope, and quadratic component of the accuracy-vs.-trial function presumably varied across participants, hence the between-subject variances of the intercept, *t*_2_, and $t_{2}^{2}$ were estimated by including ${\hat{\sigma }}_{i}^{2}$, ${\hat{\sigma }}_{t}^{2}$ and ${\hat{\sigma }}_{t^{2}}^{2}$ as three random effects in the mixed-effects model.

Equation 1 provided reasonably good fits to the data, as indicated by the best-fitting curves in Figure 3, and the residuals were distributed approximately normally and did not contain any obvious structure. Conditional *R*^2^ was 0.69. The standard deviations of the random effects are shown in Table A1. The model’s overall goodness-of-fit was reduced significantly when ${\hat{\sigma }}_{i}^{2}$, χ^2^ = 2367, df = 1, *p* < 0.001, ${\hat{\sigma }}_{t^{2}}^{2}$, χ^2^ = 58.2, df = 3, *p* < 0.001, and ${\hat{\sigma }}_{t}^{2}$, χ^2^ = 267.1, df = 3, *p* < 0.001, were removed and therefore all random effects were retained in the final version of the model.

The ANOVA table for the fixed effects is presented in Table A2. The effect of $p_{1}$ was significant because accuracy in session 2 was positively associated with accuracy on the first 21 trials of session 1. The $p_{1}\times N$ interaction was included in the model but it was not statistically significant, *F*(1, 90) = 3.46, *p* = 0.07, *f*_*p*_ = 0.20. Both the linear (*t*_2_) and quadratic ($t_{2}^{2}$) terms for session 2 trial were significant, though the effect of the linear term was nearly six times larger than the effect of the quadratic term. This result can be seen in Figure 3: accuracy increased approximately linearly across trials, but the best-fitting curves are slightly non-linear. The *N* × *T*_1_ (novelty × training) interaction was significant because the effect of training was larger in the same condition, *F*(1, 45) = 9.53, *p* = 0.003, *f*_*p*_ = 0.46, than in the novel condition (*F*(1, 45) = 0.5, *p* = 0.46, *f*_*p*_ = 0.11). This interaction can be seen in Figure 3 as an increase in the difference between accuracy in the same and novel conditions across the four panels. The novelty × training interaction also is illustrated in Figure 4a, which shows estimated accuracy at session-2 trial numbers 21 and 840 (i.e., bins 1 and 40) for each session 1 training condition, while holding the effect of $p_{1}$ constant by setting it to its mean. The interaction is shown by the difference in the slopes of the lines relating accuracy to the number of training trials. Note that this effect plot differs from Figure 2b because the novelty and training effects are shown after taking into account the estimated effects of $p_{1}$ and *t*_2_. Figure 4a suggests that accuracy in the same condition, but not the novel condition, increased proportionally with log-transformed training trials.

The novelty × training interaction shown in Figure 4a is identical at bins 1 and 40: the difference between the plotted effects for the two bins is due solely to the estimated effect of *t*_2_. The fact that estimated effects at bins 1 and 40 are simply displaced vertically implies that the effect of *t*_2_ was essentially constant across novelty and training conditions: accuracy improved with increasing session 2 trial number by approximately constant amounts in all conditions. However, this result is not surprising because the mixed-effects model defined by Equation 1 did not include a three-way interaction between *t*_2_, novelty, and training. To test whether the novelty × training interaction depended on *t*_2_, we added terms for the *t*_2_ × novelty, *t*_2_ × training, and *t*_2_ × novelty × training interactions to Equation 2. Adding these fixed effects did not improve the overall fit, χ^2^ = 0.59, df = 3, *p* = 0.89, and none of the interactions were significant, *F* ⩽ 0.24, *p* ⩾ 0.62. Hence, the novelty × training interaction did not vary significantly across session 2 trials. Overall, with respect to the effects of stimulus novelty, the results of this finer-grained analysis on accuracy throughout session 2 were consistent with the trends observed in the first 105 trials on session 2: stimulus-specific learning increased proportionally with the amount of training in session 1, and a clear effect of novelty emerged after 105 (session 1) training trials.

### Discussion

Experiment 1 extended the results of Hussain et al. (2012) by examining whether stimulus-specific learning can be obtained with fewer training trials (21 and 63), and with novel stimuli (textures). We found clear evidence of stimulus-specific learning in participants who received 840 training trials, weaker evidence in participants who received 105 training trials, and no evidence of stimulus-specific learning in participants who received 21 and 63 trials of practice (Figures 2b and 3). Thus, one way of summarizing our findings is to say that at least 105 practice trials were necessary to produce statistically significant stimulus-specific learning. However, our statistical modelling suggests an alternative summary, namely that stimulus-specific learning was a linear function of the logarithm of the number of session 1 training trials (Figure 4a), and that the difference between accuracy in the same and novel conditions was *statistically significant* (with our sample size) after approximately 105 trials. This result is consistent with the view that stimulus-specific learning occurred throughout training during session 1 (Poggio et al., 1992; Hussain et al., 2012).

Hussain et al. (2012) found that stimulus-specific learning in a face identification task was equivalent in participants who received 105 and 840 training trials. We replicated this result when we analyzed average accuracy in the first 105 trials in session 2: although the effect of novelty was slightly larger in the 840-trials condition than the 105-trials condition (see Figure 2b), the difference between conditions was not statistically significant. However, our mixed-effects analysis, which analyzed session 2 accuracy on a finer time scale, found that the effect of novelty was approximately proportional to the number of training trials (Figure 4a).

One difference between studies is that Hussain et al (2012) found more learning in session 2 in the novel conditions than in the same conditions. We did not find this effect: The novelty × *t*_2_ interaction was not significant, *F*(1, 165) = 0.22, *p* = 0.64, *f*_*p*_ = 0.04, and neither was the three-way interaction between novelty, *t*_2_, and training, *F*(1, 165) = 1.919, *p* = 0.168, *f*_*p*_ = 0.11. Hence, we found no evidence that participants in the novel groups learned more in session 2 than participants in the same groups, and the effect of stimulus novelty did not change during the course of session 2 trials. It is not clear whether this difference between studies is due to the special status of faces as stimuli, or some other factors.

In Experiment 2 we investigated whether stimulus-specific learning resulting from limited practice endures over a longer period. Our previous work had shown stimulus-specific enhancements in both face and texture identification persisted for over a year following one session of extensive practice (840 trials per session; Hussain et al., 2011). Furthermore, Aberg et al. (2009) found generalization of learning to novel stimuli after relatively smaller amounts of practice (400 trials per session) when training sessions were separated by a week. Hence, we sought to determine whether similar findings to those observed in Experiment 1 would be obtained with a 1-week gap between sessions 1 and 2.

## Experiment 2 (1-week interval)

### Methods

#### Participants

One hundred sixty-eight naïve participants, 42 males, mean age = 19.7 years, range = 17-34 years, with normal or corrected-to-normal visual acuity participated in the experiment. Other recruitment details were the same as in Experiment 1.

#### Apparatus, stimuli, and procedure

The apparatus, stimuli, and procedure were the same as those used in Experiment 1 (Figure 1A and B).

#### Design

The experimental design was the same as Experiment 1 (Figure 1C), with the exception that the two sessions were separated by 1 week, and that the number of subjects per condition differed slightly from Experiment 1. There were 48 participants each in the 1, 3, or 5 block training conditions (i.e., 21, 63, or 105 trials, respectively), and 24 participants each in the 40-block condition.^1^

### Results

The data were analyzed with the same statistical procedures used in Experiment 1.

#### Session 1: Accuracy

Accuracy on the first 21 trials in session 1 varied considerably among participants in each group, and the group means ranged from a low of 0.16 in the same-63 group to a high of 0.24 in the same-21 group (Figure 2c). However, a between-subjects ANOVA found that the effect of group was not significant, *F*(7, 160) = 1.58, *p* = 0.14, *f* = 0.26. Analyzing the data with a 2 (novelty) × 4 (training) ANOVA also yielded no significant effects, *F* ⩽ 1.78, *p* ⩾ 0.15, *f* ⩽ 0.18. Hence, we did not find a statistically significant difference across groups at baseline.

#### Session 2: Accuracy, the first 105 trials

Accuracy during the first 105 trials in session 2 is plotted in Figure 2d. As in Experiment 1, accuracy in all conditions was greater than baseline (i.e., accuracy in the first 21 trials in session 1). Also, accuracy in the same condition, but not the novel condition, increased monotonically with the number of training trials. There also is the suggestion of a non-linear effect of training, with session 2 accuracy increasing suddenly when session 1 practice exceeded 63 trials.

A 2 (novelty) × 4 (training) ANOVA found significant main effects of novelty, *F*(1, 160) = 5.46, *p* = 0.021, *f*_*p*_ = 0.18, and training, *F*(3, 160) = 7.18, *p* < 0.001, *f*_*p*_ = 0.37, and a non-significant novelty × training interaction, *F*(3, 160) = 2.18, *p* = 0.09, *f*_*p*_ = 0.20. Planned trend analyses found that the linear trend of accuracy across log-transformed training trials was significant and differed between the novelty conditions: the trend was significant in the same condition, *F*(1, 80) = 17.81, *p* < 0.0001, *f* = 0.51, but not in the novel condition, *F*(1, 80) = 1.42, *p* = 0.24, *f* = 0.24. Also, the nonlinear trends were not significant, *F*(2, 160) = 2.77, *p* = 0.066, *f*_*p*_ = 0.19, and did not differ significantly between novelty conditions, *F*(2, 160) = 0.20, *p* = 0.816, *f*_*p*_ = 0.05. These trend analyses suggest that average accuracy was approximately proportional to the log-transformed number of training trials in the same condition but not the novel condition, and therefore that the difference between accuracy in the same and novel conditions increased with training trials. The simple main effect of novelty was significant in the 840-trials, *F*(1, 160) = 6.25, *p* = 0.013, *f*_*p*_ = 0.20, and 105-trials, *F*(1, 160) = 4.04, *p* = 0.046, *f*_*p*_ = 0.16, conditions, but not in the 63-trials, *F*(1, 160) = 1.38, *p* = 0.242, *f*_*p*_ = 0.09, and 21-trials, *F*(1, 160) = 0.337, *p* = 0.562, *f*_*p*_ = 0.05, conditions. Therefore, as was found in Experiment 1, stimulus-specific learning occurred with 105 practice trials, and the trend analyses suggested an even earlier onset of such learning.

As was done in Experiment 1, we examined whether stimulus-specific learning differed between participants who received 105 and 840 training trials by analyzing the data from the 105-trials and 840-trials conditions with a 2 (novelty) × 2 (training) ANOVA. The main effect of novelty was significant, *F*(1, 68) = 7.96, *p* < .01, *f* = 0.34, but the main effect of training, *F*(1, 68) = 0.89, *p* = 0.0.35, *f* = 0.11, and the novelty × training interaction, *F*(1, 68) = 0.65, *p* = 0.42, *f* = 0.10, were not. Thus, we failed to find evidence that the effect of novelty differed between the 105-trials and 840-trials conditions.

#### Session 2: Time course and individual differences

Accuracy in session 2, averaged across participants, is plotted for each bin in Figure 5. In all conditions, accuracy increased approximately linearly with the logarithm of the number of trials in session 2, although the slope of the accuracy-vs.-trials function was noticeably lower in the 840-trials condition than the other conditions. In conditions in which participants received 21 training trials in session 1, there was no evidence of stimulus-specific learning in session 2. In fact, accuracy was slightly higher in the novel condition than the same condition near the end of session 2. In conditions in which participants received 63 training trials in session 1, accuracy was slightly higher in the same condition than the novel condition during the first ≈100 session 2 trials, but not during subsequent trials. In the 105 and 840 training trials conditions, accuracy was higher in the same condition than the novel condition throughout session 2. With respect to accuracy in the last bin of session 1, accuracy in the first bin of session 2 showed essentially no decline for the same groups, but an increasingly larger decline with amount of session 1 training for the novel groups. This result is consistent with what was observed after a day’s delay (Experiment 1). A more detailed view of the time course across sessions is provided in Figure S1 (Supplementary material). That figure shows there was a slight decrease in accuracy in the same condition between the last block in session 1 and the first block in session 2, and this decrease was larger in groups receiving 105 and 840 training trials. However, in the novel condition there was a larger decrease in accuracy between session 1 and session 2, particularly in the 840-trials condition.

As in Experiment 1, the data were analyzed with a linear mixed-effects model with the same fixed predictor variables as described for that experiment. Again, we used a model-comparison approach to establish the simplest model, with the final fixed-effects model defined by the equation

(2) $$\begin{array}{ccc} y & \;= & \mu_{0}+p_{1}+N+T_{1}+t_{2}+t_{2}^{2}\\ & & +\;\left(p_{1}\times T_{1}\right)+\left(N\times T_{1}\right)+\left(T_{1}\times t_{2}\right)\; \end{array}$$

where *y* is proportion correct in a 21-trial bin in session 2, μ_0_ is the intercept, $p_{1}$ is proportion correct on the first 21 trials in session 1, *N* is stimulus novelty in session 2, *T*_1_ is log-transformed session 1 training trials, and *t*_2_ is log-transformed session 2 trial. Note that this model differs from the one defined by Equation 1. Finally, the model allowed the intercept, slope, and quadratic component of the accuracy-vs.-trial function to vary across participants by including ${\hat{\sigma }}_{i}^{2}$, ${\hat{\sigma }}_{t}^{2}$ and ${\hat{\sigma }}_{t^{2}}^{2}$ as three random effects.

The model provided reasonably good fits to the averaged data, as indicated by the best-fitting curves in Figure 5, and the residuals were distributed approximately normally and did not contain any obvious structure. Conditional *R*^2^ (Bartoń, 2022) was 0.67. The standard deviations of the random effects were similar to those found in Experiment 1 (Table A1). The model’s overall goodness-of-fit was reduced significantly when ${\hat{\sigma }}_{i}^{2}$, χ^2^ = 4368, df = 1, *p* < 0.001, ${\hat{\sigma }}_{t^{2}}^{2}$, χ^2^ = 46.5, df = 3, *p* < 0.001, and ${\hat{\sigma }}_{t}^{2}$, χ^2^ = 284.5, df = 3, *p* < 0.001, were dropped and therefore all three random effects were retained in the final version of the model.

The ANOVA table for the fixed effects is presented in Table A3. The coefficients for the covariate ($p_{1}$) and the $p_{1}\times T_{1}$ interaction were significant: Overall, there was a positive association between $p_{1}$ and accuracy, but the association varied across training conditions. Specifically, the estimated effect of $p_{1}$, represented by the slope of the line relating $p_{1}$ and session 2 accuracy, increased with the log-transformed number of training trials: the slope was 2.15 times greater in the 840 trials condition (*m* = 0.82) than in the 21 trials condition (*m* = 0.38). One way of describing this interaction is to say that participants who performed best initially in session 1 tended to perform best in session 2, and this trend was stronger in participants who received lots of training trials in the first session. In other words, training seemed to reinforce or enhance the association between initial accuracy and session 2 performance.

The fixed effect coefficients for both *t*_2_ and $t_{2}^{2}$ also were significant, although, as in Experiment 1, the effect size for the linear term was 5.6 times larger than the effect size for the quadratic term. This result can be seen in Figure 5: accuracy increased approximately linearly across trials, but the best-fitting curves are slightly non-linear. The *t*_2_ × *T*_1_ interaction was significant because the effect of session 2 trials differed across training conditions: The linear trend of estimated accuracy (averaged across novelty conditions) across log-transformed session 2 trials was largest in the 21 trial condition, *f*_*p*_ = 1.39, smallest in the 840 trials, condition, *f*_*p*_ = 0.78, and intermediate in the 63 trial, *f*_*p*_ = 1.29, and 105 trial, *f*_*p*_ = 1.20, conditions. Another way of stating this result is that accuracy in the four training conditions (averaged across novelty conditions) differed noticeably at the start of session 2 but was nearly equal by the end of session 2. We return to this point in the following section when we analyze the two experiments with a single model.

As in Experiment 1, the mixed-effects model analysis found that the novelty × training (*N* × *T*_1_) interaction was significant because accuracy increased with trial number faster in the same condition than the novel condition: the effect of training was significant in the same condition, *F*(1, 83) = 4.81, *p* = 0.031, *f*_*p*_ = 0.24, but not the novel condition, *F*(1, 82) = 1.83, *p* = 0.126, *f*_*p*_ = 0.17. This result is consistent with what was found with average accuracy in the first 105 trials in session 2 (Figure 2d). The novelty × training interaction can be seen in Figure 5 as an increase in the difference between average accuracy in the same and novel conditions as training trials increase (across panels) from 21 to 840. The interaction also is illustrated in the effect plot in Figure 4b, which shows the interaction at the start and end (i.e., bins 1 and 40) of session 2. Inspection of Figure 4b shows that the difference between accuracy in the same and novel conditions increased with increasing training trials, and that this increase in the same/novel difference across training trials was the same at bin 1 and bin 40.

In summary, when session 2 occurred after a week’s delay, we found clear evidence of stimulus-specific learning in participants who received 105 and 840 trials of practice, and no evidence of stimulus-specific learning in participants who received 21 and 63 trials of practice. Thus, as was the case in Experiment 1, one way of summarizing our findings is to say that at least 105 practice trials were necessary to produce statistically significant stimulus-specific learning, suggesting that some criterion amount of practice is needed to produce stimulus-specific learning. However, as in Experiment 1, we found that effect of stimulus novelty increased as a linear function of the logarithm of the number of session 1 practice trials (Figure 4b). This result is consistent with the view that stimulus-specific learning occurred throughout training during session 1 (Poggio et al., 1992; Hussain et al., 2012), and that the difference between accuracy in the same and novel conditions was statistically significant (with our sample size) after approximately 105 trials.

## Comparison of Experiments 1 and 2

In many respects the analyses of Experiments 1 and 2 yielded similar results. In both experiments, accuracy in session 2 was related to initial accuracy in session 1 ($p_{1}$), the amount of training in session 1, and stimulus novelty. More specifically, after statistically accounting for variation in $p_{1}$, the difference between response accuracy in the same and novel conditions increased linearly with log-transformed training trials. Indeed, the novelty × training interaction measured at the start of session 2 was very similar in the two experiments (Figure 4), which suggests that the effects of practice did not depend on the length of the retention interval (i.e., one day vs. one week). However, the analyses also found that the effect of $p_{1}$ (initial accuracy), on session 2 accuracy increased with the number of session 1 training trials when the sessions were separated by one week (Experiment 2) but not when they were separated by one day (Experiment 1). Also, after a 1-week interval but not after a 1-day interval, the effect of session 1 training trials declined with session 2 trial number, which implies that the beneficial effects of additional training in session 1 faded over the course of session 2 when the sessions were separated by 1 week.

Differences between the results obtained in the two experiments were evaluated quantitatively in three ways. First, we analyzed the data in Figures 2b and d with a 2 (novelty) × 4 (training) × 2 (experiment) ANOVA. The linear trend of accuracy across log-transformed training trials was significant, *F*(1, 248) = 30.49, *p* < 0.001, *f* = 0.35, and differed between novelty conditions, *F*(1, 248) = 12.77, *p* < 0.001, *f* = 0.23. None of the other effects were significant, *F* ⩽ 1.21, *p* ⩾ 0.29, *f* ⩽ 0.07. Follow-up *t* tests found that the effect of novelty was significant in the 840 trials, *t*(43.3) = 3.96, *p* < 0.001, *d* = 1.20, and 105 trials, *t*(69.9) = 2.49, *p* = 0.015, *d* = 0.60, conditions, but not in the 63 trials, *t*(59.9) = 0.12, *p* = 0.12, *d* = 0.41, and 21 trials, *t*(58.7) = −0.64, *p* = 0.53, *d* = −0.17, conditions. These results are very similar to the ones obtained by the ANOVAs performed on the individual experiments.

Next, we analyzed the proportion correct in the first block of trials (i.e., bin 1) in session 2 with a linear model that included proportion correct in the first 21 trials of session 1 (*p*_1_), log-transformed session 1 training trials (*T*_1_), and two binary factors representing novelty and experiment. The effects of *p*_1_, *F*(1, 255) = 41.13, *p* < 0.0001, *f* = 0.40, and *T*_1_, *F*(1, 255) = 22.32, *p* < 0.0001, *f* = 0.30, were significant. The main effect of novelty was not significant (*F*(1, 255) = 3.52, *p* = 0.062, *f* = 0.12), but the novelty × *T*_1_ interaction (*F*(1, 255) = 9.03, *p* < 0.003, *f* = 0.19) was significant. None of other effects were significant, *F* < 1, *p* > 0.37, *f* ⩽ 0.04. Follow-up analyses found that the effect of *T*_1_ was significant in the same condition, *F*(1, 127) = 25.01, *p* < 0.001, *f* = 0.44, but not in the novel condition, *F*(1, 127) = 1.75, *p* = 0.19, *f* = 0.12. To examine the degree to which accuracy in the same condition was a linear function of log-transformed training trials, we added a quadratic coefficient ($T_{1}^{2}$) to the model. The quadratic coefficient’s effect size was small and not statistically significant, *F*(1, 128) = 0.013, *p* = 0.91, *f* = 0.01. Thus, this analysis found that the effect of stimulus novelty on accuracy in the first block of session 2 trials increased approximately linearly with logarithm of the number of session 1 training trials and that the effect of novelty did not differ significantly between experiments.

### Session 2: Time course

Finally, we analyzed the data in Figures 3 and 5 with a linear mixed-effects model that included all of the fixed effects listed in Equations 1 and 2 plus a two-level factor representing experiment that was allowed to interact with all of the other fixed effects. The model also included ${\hat{\sigma }}_{i}^{2}$, ${\hat{\sigma }}_{t}^{2}$, and ${\hat{\sigma }}_{t^{2}}^{2}$ as random, within-subject effects. The standard deviations of the random effects were similar to those found in the separate analyses of the two experiments (Table A1), and all three were statistically significant, *p* < 0.001.

The ANOVA table for the fixed effects is shown in Table A4. As was found in the separate analyses of Experiments 1 and 2, the effect of *t*_2_ (trial number in session 2), was more than five times larger than the effect of $t_{2}^{2}$, indicating that accuracy during session 2 increased approximately linearly with log-transformed trial number. However, the effect of *t*_2_ varied significantly across training conditions and experiments (Figures 6a and b). When the sessions were separated by one day the effect of training persisted throughout session 2 and the *t*_2_ × *T*_1_ interaction (session-2 trial number by session-1 training), was not significant, *F*(1, 94) = 0.09, *p* = 0.76, *f*_*p*_ = 0.03. On the other hand, when the sessions were separated by 1 week, the effect of session 1 training trials (*T*_1_) diminished with increasing session 2 trial number (*t*_2_), and the *t*_2_ × *T*_1_ interaction was significant, *F*(1, 166) = 21.65, *p* < 0.001, *f*_*p*_ = 0.36. This three-way interaction suggests that the effect of training persisted throughout session 2 when the sessions were separated by one day but not one week (Figure 6). Alternatively, the interaction shows that extensive practice in session 1 improved performance at the start of session 2 in both experiments, but improved performance at the end of session 2 only when the sessions were separated by one day.

### Individual differences

The combined analysis also found that accuracy in the first block of session 1 ($p_{1}$) was positively associated with accuracy in session 2 in all conditions in both experiments. The slope of the line relating *p*_1_ and accuracy in session 2 was steeper in the same condition than the novel condition, but neither the $p_{1}\times \mathrm{novelty}$ interaction, *F*(1, 250) = 3.74, *p* = 0.054, *f*_*p*_ = 0.12, nor the $p_{1}\times \mathrm{novelty}\times \mathrm{experiment}$ interaction, *F*(1, 250) = 0.29, *p* = 0.59, *f*_*p*_ = .03, were significant. Thus, the effect of novelty did not vary significantly with initial session 1 performance. However, the $p_{1}\times T_{1}\times \mathrm{experiment}$ interaction was significant, *F*(1, 250) = 5.09, *p* = 0.025, *f*_*p*_ = 0.14: The effect of $p_{1}$ (i.e., the slope of the line relating $p_{1}$ and session 2 accuracy) varied significantly across training conditions and experiments. Specifically, the combined analysis found that the slope of the line relating accuracy in the two sessions was 2.25 times greater in the 21 trials condition than the 840 trials condition when the sessions were separated by one day, but was 2.1 times *smaller* in the 21 trials condition than in the 840 trials condition when the sessions were separated by 1 week. This interaction is illustrated by the effects plots in Figures 7a and b, which show estimated accuracy, averaged across novelty conditions, for the first 21 trials in session 2 (also see Figure S2). In both plots, session 2 accuracy is positively associated with initial session 1 performance ($p_{1}$): Participants who performed well initially in session 1 tended to perform well initially in session 2. However, the slopes of the lines decrease with training trials in Experiment 1 (1-day interval) but *increase* with training in Experiment 2 (1-week interval). Thus, after one day, training had its largest effect on participants who initially performed poorly, but after one week, training had its largest effect on participants who initially performed well.

The effects plots in Figures 7c and d show estimated accuracy, averaged across novelty conditions, for the *last* 21 trials in session 2. A comparison of top and bottom rows of Figure 7 shows that the lines for each training condition are shifted vertically but that the slopes are unchanged. The vertical shift reflects the effect of session 2 trial number (*t*_2_): accuracy increased from bin 1 to bin 40, and so estimated accuracy is higher in Figures 7c and d than 7a and b. When the sessions were separated by one day, the effect of *t*_2_ did not vary across training conditions (Figure 6a), so the lines in Figures 7b and d are shifted vertically by nearly identical amounts. Therefore, after a day’s delay the effect of training at the end of session 2, like the effect at the start of session 2, was greatest among poor performers and nearly zero among good performers (Figure 7c). However, when the sessions were separated by one week the effect of *t*_2_ varied across training conditions (Figure 6b), and therefore the lines in Figures 7a and c are shifted by different amounts. Consequently, in Experiment 2 accuracy at the end of session 2 was positively associated with the number of session 1 training trials in good performers, but negatively associated with training in poor performers (Figure 7d).

### Stimulus novelty

Finally, the mixed-effects model found significant main effects of stimulus novelty (*N*) and log-transformed number of training trials (*T*_1_), and a significant *N* × *T*_1_ interaction. The interaction was significant because the effect of training was larger in the same condition, *F*(1, 130) = 12.23, *p* < 0.001, *f*_*p*_ = 0.31, than the novel condition, *F*(1, 127) = 0.19, *p* = 0.666, *f*_*p*_ = 0.04. The novelty × training interaction did not differ significantly between experiments, *F* < 1, *p* = 0.62, *f*_*p*_ = 0.03. Our separate analyses of Experiments 1 and 2 found that the *t*_2_ × novelty × training interaction was not significant. Nevertheless, it is possible that that three-way interaction could vary between experiments. We tested this idea by re-analyzing the combined data with a mixed-effects model that included terms for the *t*_2_ × novelty, *t*_2_ × novelty × training, and *t*_2_ × novelty × training × experiment interactions. Adding these fixed effects did not change the overall goodness-of-fit significantly, χ^2^ = 2.07, df = 3, *p* = 0.56, and none of the interactions were significant, *F* ⩽ 1.79, *p* ⩾ 0.18, *f*_*p*_ ⩽ 0.08. So the novelty × training interaction did not vary significantly across session 2 trials or experiments.

In summary, the combined analyses found that Experiments 1 and 2 differed in terms of the effect of the amount of session 1 training and the effect of initial session 1 performance, but not in the effect of stimulus novelty. The number of session 1 training trials produced differences in performance on session 2 that were nearly constant throughout the session when the sessions were separated by one day, but which faded away during session 2 when the sessions were separated by a week (Figure 6). Additionally, training affected good and poor performers differently in the two experiments. When the sessions were separated by one day, the largest benefits of training were for participants who performed poorly in the first block of trials in session 1; however, when the sessions were separated by 1 week the largest benefits of training were for participants who performed the best in the first block of session 1 (Figure 7). Importantly, we found no evidence that the novelty × training interaction differed between experiments. Regardless of whether the sessions were separated by 1 day or 1 week, the difference between accuracy in the same and novel conditions was proportional to the logarithm of the number of session 1 training trials, and the difference was statistically significant after approximately 105 trials.

## General discussion

The current experiments investigated perceptual learning by measuring response accuracy in a 1-of-10 texture identification task across two sessions separated by one day (Experiment 1) or one week (Experiment 2). Performance in session 2 was influenced by several factors, including i) initial performance in session 1; ii) the number of session 1 training trials; iii) the session 2 trial number; and iv) whether the stimuli in session 2 were novel. We found that, after statistically accounting for the other factors that affected performance, the difference between accuracy in session 2 in the same and novel conditions increased linearly with the log-transformed number of session 1 training trials. This result is consistent with the hypothesis that stimulus-specific perceptual learning begins very early in practice, perhaps in the first few trials, and becomes statistically significant later in practice at a point that depends on the statistical power of the experiment. Here, the difference between the same and novel groups was statistically significant after approximately 100 practice trials, as was found with a face identification task (Hussain et al., 2012), suggesting that rapid learning of stimulus properties characterizes pattern identification more generally, and not face identification specifically. Furthermore, the effect of stimulus novelty did not differ between inter-session intervals (Cf. Figures 4a and b), and did not vary across session 2 trial number. Therefore, rapid stimulus-specific improvements in the current task were relatively long lasting and robust to further training during the test session. Unlike some previous studies, participants in our experiments were not given preliminary practice and no trials were discarded. Thus, stimulus-specific information seems to be learned at first exposure to the task. It has been suggested that steeper increases in performance early in the time course of learning are associated with learning of general aspects of the task, and more gradual, later improvements are due to learning of stimulus-specific properties (e.g., Karni & Bertini, 1997; Karni et al., 1998). However, we found little evidence for distinct, sequential phases in learning. Instead, the similarity of slopes of the learning curves in session 2 in the same and novel conditions suggest that stimulus-specific and general learning occurred concurrently throughout practice.

### Comparison with previous work

Jeter et al. (2010) found more generalization of learning of orientation discrimination to untrained retinal locations and stimulus orientations after relatively few practice trials (1,200 vs. 7,000+ trials). Aberg et al. (2009) found more generalization of learning in a hyperacuity task when 1,600 trials were distributed over 4 days (400 trials/day) than over two days (800 trials/day). We did find that after a 1-week interval (Experiment 2), accuracy for novel stimuli at the end of session 2 was negatively (albeit non-significantly) associated with the number of session 1 training trials (Figure 4b). However, accuracy for novel stimuli at the start of session 2 was slightly positively associated with the number of practice trials. Also, after a one day interval (Experiment 1), accuracy in the novel condition was essentially independent of the number of practice trials throughout session 2 (Figure 4a). Therefore, we did not find strong evidence that extensive practice reduced generalization of learning to novel stimuli in our task. Of course, the current experiments differed in several ways from those reported by Jeter et al. (2010) and Aberg et al. (2009). For example, Jeter et al. (but not Aberg et al.) used an adaptive staircase to vary stimuli across trials whereas the current experiments used the method of constant stimuli. The studies also used different stimuli and tasks, and differed in the way practice was distributed within and across sessions. Additional work is needed to understand how those methodological differences contributed to the different results.

### Other predictors of learning: Amount of practice and individual differences

In both experiments, the strongest predictor of performance in session 2 was the log-transformed trial number (*t*_2_ effect size: 1.24 and 1.23), followed by individual differences at baseline ($p_{1}$ effect size: 0.50 and 0.62). Average accuracy increased approximately linearly against log trial for all groups, consistent with a single exponential function for perceptual learning as shown for various tasks (Dosher & Lu, 2007; Hussain et al., 2009b; Zhang, Zhao, Dosher, & Lu, 2019; Cochrane & Green, 2021; Yang et al., 2022). Stimulus novelty significantly affected the intercept but not the slope of the accuracy-vs.-trial function (Figures 3 and 5), but the effect size for novelty (*N* = 0.3 and 0.14) was smaller than the effects of *t*_2_ and $p_{1}$. In the following sections we discuss the contribution of training and individual differences to learning of our task.

### Amount of practice

We found that the slope of the learning curve in session 2 depended on the number of session 1 training trials when the two days were separated by 1 week but not when they were separated by 1 day (Figure 6). One day after training, session 2 accuracy increased at the same rate against trial number for all groups, so performance at the end of session 2 remained ordered by the amount of session 1 practice (Figure 6a). On the other hand, a week after training the slopes of the learning curves were shallower for groups that received more training trials in session 1, so performance in the last few trials in session 2, averaged across novelty conditions, was nearly equal across groups (Figure 6b). Hence, the benefits of additional practice were observed at the start of session 2 in both experiments, but were observed at the end of session 2 only in Experiment 1 (after a 1-day interval). This result, combined with the finding that the *N* × *T*_1_ did not differ between experiments, suggests that the longer 1-week interval between sessions constrained the amount of learning (or the retention of learning) by similar amounts in the same and novel conditions. Between-session forgetting is characteristic of some types of perceptual learning (Yang et al., 2022), but it is not clear how it depends on the training-test interval. Based on the current results, we predict that increasing the training-test interval beyond 1 week would further diminish the benefits of large amounts of practice, but would not affect the stimulus-specificity of learning.

### Individual differences

Initial accuracy in session 1 was a strong predictor of performance in session 2 (e.g., Hussain et al., 2011; Yang et al., 2020), even when the two sessions were separated by 1 week. This result suggests that individual differences were at least somewhat stable across sessions, and is consistent with previous studies finding stable individual differences in higher-level object recognition, visual attention, and gaze (Sheikh et al., 2014; Andermane, Bosten, Seth, & Ward, 2019; de Haas, Iakovidis, Schwarzkopf, & Gegenfurtner, 2019; Richler et al., 2019; Wilbiks & Beatteay, 2020; Veríssimo, Hölsken, & Olivers, 2021). In this sense, our results are consistent with the hypothesis that individual differences in general perceptual or recognition abilities may be linked to differences in cortical morphology or dynamics (e.g., Eayrs & Lavie, 2019; Stacchi, Liu-Shuang, Ramon, & Caldara, 2019) and associated with different learning strategies (Forest, Siegelman, & Finn, 2022; Smithson, Eichbaum, & Gauthier, 2023). Our mixed-effects model analyses found a significant association between initial performance and the intercept of the session 2 accuracy-vs.-trial function, but not its slope. This means that the rate of within-session learning in session 2 did not depend on initial performance, in contrast with what has been found in some studies (Fahle & Henke-Fahle, 1996; Yang et al., 2020). Our mixed-effects model analyses also found that the intercept, slope, and curvature of the session 2 learning curve varied significantly across individuals, and that the size of these random effects were large. In other words, the learning curves in session 2 varied significantly across participants. This result is consistent with previous reports that perceptual learning varies considerably across individuals (Fine & Jacobs, 2002; Jeter et al., 2010). Future work examining learning curves across multiple days will help to determine the extent to which these random effects represent stable individual differences in learning.

### Interactions between amount of practice and individual differences

Our results suggest that the effect of session 1 training depended on how well participants performed in the first block of session 1, and that this dependency differed between experiments (Figure 7). In Experiment 1, the estimated effects of session 1 training were largest among participants who initially performed poorly in session 1 (Figure 7a). Furthermore, accuracy in session 2 improved by similar amounts in all training conditions (Figure 6a), and therefore at the end of session 2 the effects of training were still largest among participants who initially performed poorly in session 1 (Figure 7b). In Experiment 2, unlike Experiment 1, the effects of session 1 training were largest among participants who performed well initially in session 1 (Figure 7b). In other words, good performers benefited most from session 1 training. However, accuracy in session 2 improved more in the 21-trials condition than the 840-trials condition (Figure 6b), and therefore by the end of session 2 the effect of training among good performers was reduced and among poor performers there was a small *negative* association between accuracy and training (Figure 7d).

One way of summarizing these results is to say that when the sessions were separated by one day, training during session 1 benefited poor performers more than good performers, and this benefit of practice persisted throughout session 2. However, when the sessions were separated by one week, training during session 1 benefited good performers but practice in session 2 allowed poor performers to catch up, perhaps because accuracy among good performers approached an upper limit (Figure 6b). This difference between experiments suggests that poor performers can benefit from extensive training, but that some of the benefits of practice are diminished following a one week delay between training and test sessions. The results obtained with good performers are more puzzling. One could reasonably expect good performers to benefit from practice and then reach an upper limit, as they did in Experiment 2. However, it is not obvious why training had no effect on performance among good performers in Experiment 1. One might argue that brief practice in session 1 was sufficient to allow good performers to reach an upper limit on accuracy, but this idea is inconsistent with the observation that response accuracy among good performers improved significantly during session 2 (Cf. Figures 7a and c). So it remains unclear why the effect of training was so small among good performers in Experiment 1.

### Summary

Overall, our results are consistent with past research showing statistically-reliable stimulus-specific learning early in training (e.g., Wright & Fitzgerald, 2001; Hawkey et al., 2004; Hussain et al., 2012). However, our results further suggest that, at least in some circumstances, stimulus-specific learning occurs throughout practice, perhaps concurrently with general learning. In addition, we found that the stimulus-specific effects of a small number of practice trials are similar after retention intervals of one day and 1 week, which extends previous reports that 840 practice trials produces long-lasting, stimulus-specific perceptual learning (e.g., Hussain et al., 2011). Finally, our results suggest that the initial baseline differences in task performance modulate the effects of practice in a manner that depends on the interval between practice and test sessions.

### Conclusions

Stimulus-specific effects occur very early in learning, and are relatively enduring even when produced by small amounts of practice. We found limited evidence for sequential phases of general and stimulus-specific learning. Instead, our results suggest that both types of learning, at least in these types of identification tasks, occur concurrently throughout practice. We interpret our results to suggest that the various processes governing perceptual learning are engaged at the beginning of practice and do not change qualitatively across the first several hundred trials of practice. Future research on perceptual learning should examine whether stimulus-specific learning occurs on a continuum, rather than testing solely for the presence or absence of stimulus-specific learning.

## Supplementary Material

Supplement 1

## Footnotes

1 We tested more participants in the 21, 63, and 105 trials conditions because these were novel conditions for which the effects were unknown, whereas previous studies (Hussain et al., 2009b; Hussain, Sekuler, & Bennett, 2009c; Hussain et al., 2012) using similar stimuli and procedures have already shown that 840 trials produces significant stimulus-specific learning that lasts over intervals of 1 day and 1 year.

## Appendix: Statistical tables

**Table A1.** Standard deviations of the random effects in the mixed-effects models used to analyze Experiments 1 and 2 and the combined experiments.

	${\hat{\sigma }}_{i}$	${\hat{\sigma }}_{t_{2}}$	${\hat{\sigma }}_{t_{2}^{2}}$	Residual
1-day experiment	0.1107	0.1234	0.0837	0.0973
1-week experiment	0.1136	0.1041	0.0663	0.1016
Combined	0.1126	0.1115	0.0728	0.1000

**Table A2.** ANOVA table for linear mixed-effects model (Eq. 1) fit to accuracy data from the 1-day interval experiment (Figure 3). *N* is a two-level factor representing stimulus novelty in session 2, *t*_2_ represents log-transformed session 2 trial number (i.e., 21, 42, 63, ⋅⋅⋅, 840), *T*_1_ is log-transformed session 1 training trials, and $p_{1}$ is accuracy on the first 21 trials in session 1. The table shows partial Cohen’s *f* (*f*_*p*_) as well as type III sums of squares (Sum Sq) and numerator (DF_1_) and denominator (DF_2_) degrees of freedom calculated with Satterthwaite’s method (Satterthwaite, 1946).

	Sum Sq	DF_1_	DF_2_	F	Pr(>F)	*f*_*p*_
$p_{1}$	0.2127	1	90	20.449	<0.001*	0.50
*N*	0.0775	1	90	8.180	0.005*	0.30
*T*_1_	0.1984	1	90	20.942	<0.001*	0.48
*t*_2_	1.3945	1	95	147.176	<0.001*	1.24
$t_{2}^{2}$	0.0381	1	95	4.021	0.048*	0.21
$p_{1}\times N$	0.0328	1	90	3.459	0.066	0.20
*N* × *T*_1_	0.1037	1	90	10.942	0.001*	0.35

**Table A3.** ANOVA table for the linear mixed-effects model (Eq. 2) fit to accuracy data from the 1-week interval experiment that are plotted in Figure 5. Symbols are the same as in Table A2.

	Sum Sq	DF_1_	DF_2_	F	Pr(>F)	*f*_*p*_
$p_{1}$	0.6334	1	162	61.388	<0.001*	0.62
*N*	0.0305	1	162	2.959	0.087	0.14
*T*_1_	0.0085	1	163	0.829	0.364	0.07
*t*_2_	2.5825	1	167	250.295	<0.001*	1.23
$t_{2}^{2}$	0.0827	1	167	8.011	0.005*	0.22
$p_{1}\times T_{1}$	0.0438	1	162	4.243	0.041*	0.16
*N* × *T*_1_	0.0527	1	162	5.107	0.025*	0.18
*t*_2_ × *T*_1_	0.2234	1	166	21.651	<0.001*	0.36

**Table A4.** ANOVA table for the fixed effects of a linear mixed-effects model fit to the combined data from Experiments 1 and 2. *E* stands for a two-level factor representing experiment. Other symbols are the same as in Tables A2 and A3.

	Sum Sq	DF_1_	DF_2_	F	Pr(>F)	$f_{P}$
$p_{1}$	0.7128	1	250	71.202	<0.001*	0.53
*N*	0.0881	1	250	8.795	0.003*	0.19
*T*_1_	0.0599	1	257	5.979	0.015*	0.15
*t*_2_	3.8762	1	274	387.183	<0.001*	1.19
$t_{2}^{2}$	0.1204	1	263	12.027	0.001*	0.21
*E*	0.0239	1	257	2.383	0.124	0.10
$p_{1}$ ×*N*	0.0375	1	250	3.742	0.054	0.12
$p_{1}$ ×*T*_1_	0.0022	1	250	0.225	0.636	0.03
*N* × *T*_1_	0.1391	1	250	13.891	<0.001*	0.24
*t*_2_ × *T*_1_	0.0995	1	260	9.942	0.002*	0.20
$p_{1}$ × *E*	0.0194	1	250	1.934	0.166	0.09
*N* × *E*	0.0036	1	250	0.359	0.550	0.04
*t*_2_ × *E*	0.0477	1	260	4.763	0.030*	0.14
*T*_1_ × *E*	0.0231	1	257	2.312	0.130	0.09
$p_{1}$ × *T*_1_ × *E*	0.0509	1	250	5.089	0.025*	0.14
$p_{1}$ × *N* × *E*	0.0029	1	250	0.289	0.591	0.03
*N* × *T*_1_ × *E*	0.0024	1	250	0.240	0.624	0.03
*t*_2_ × *T*_1_ × *E*	0.0711	1	260	7.103	0.008*	0.17
