# Supplementary material for: The time course of stimulus-specific perceptual learning
Source: J Vis. 2024 Apr 11;24(4):9. doi: 10.1167/jov.24.4.9 (PMC11019584; doi:10.1167/jov.24.4.9)
Supplement: Supplement 1 [file jovi-24-4-9_s001.pdf]

The time course of stimulus-specific perceptual learning
Supplementary Material

Bennett, PJ, Hashemi, A, Lass, JW, Sekuler, AB, & Hussain, Z

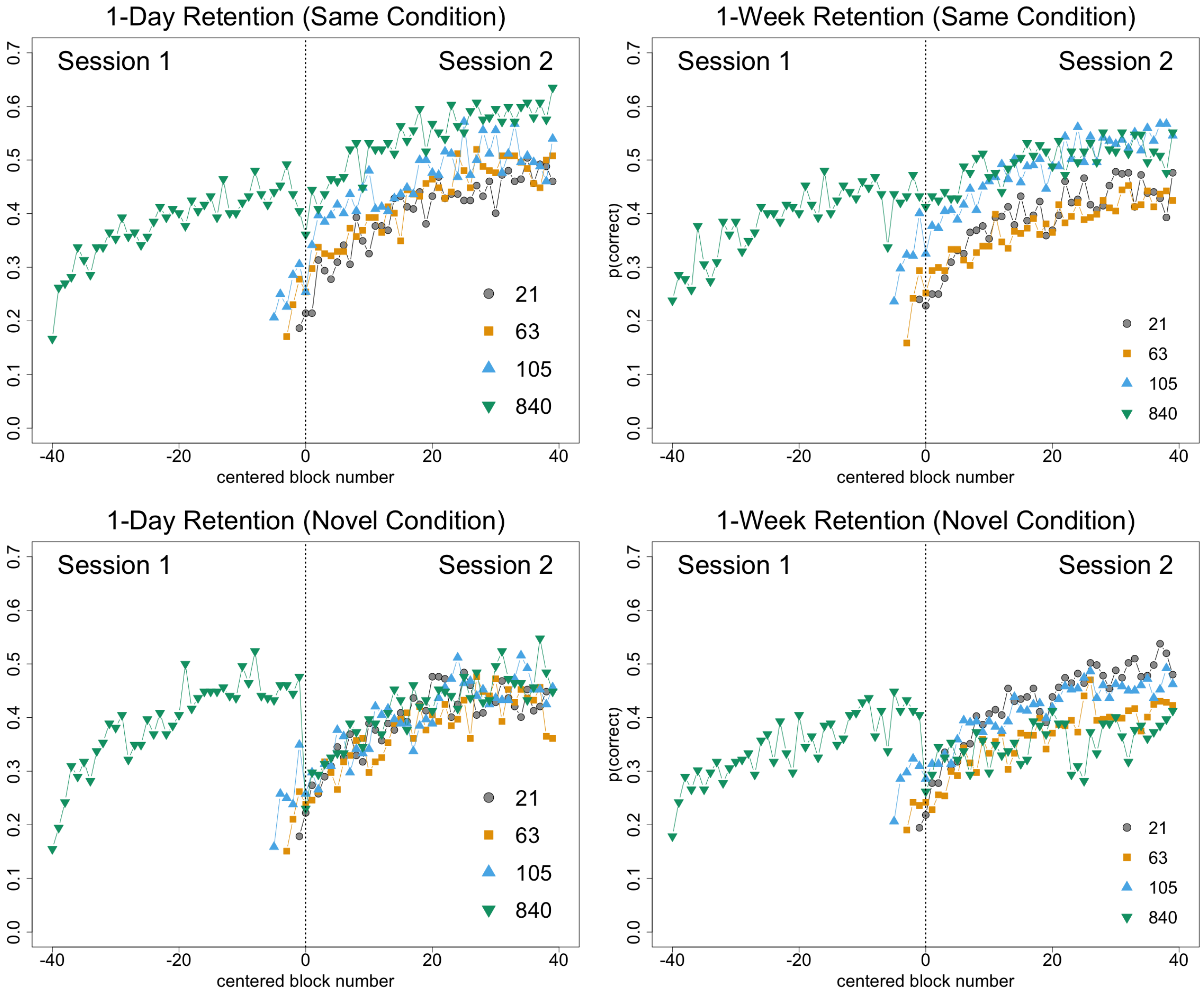

Figure S1: Response accuracy in Experiments 1 (left) and 2 (right) in the Same (top) and Novel (bottom) conditions. Each point is proportion correct in a 21-trial block: The first block in Session 2 was defined as block zero: negative block numbers occurred during Session 1 and positive block numbers occurred during Session 2. The data from different training conditions have been aligned on block zero. In the Same condition (top row), there was a slight decrease in accuracy between the last block in Session 1 and the first block in Session 2, and this decrease was larger in groups receiving 105 and 840 training trials. In Session 2, accuracy was highest in the 840-trials condition and lowest in the 21-trial condition. In the Novel condition (bottom row), there was a large drop in accuracy between the last block in Session 1 and the first block in Session 2, particularly in the 840-trials condition, and accuracy in Session 2 was similar across training conditions.

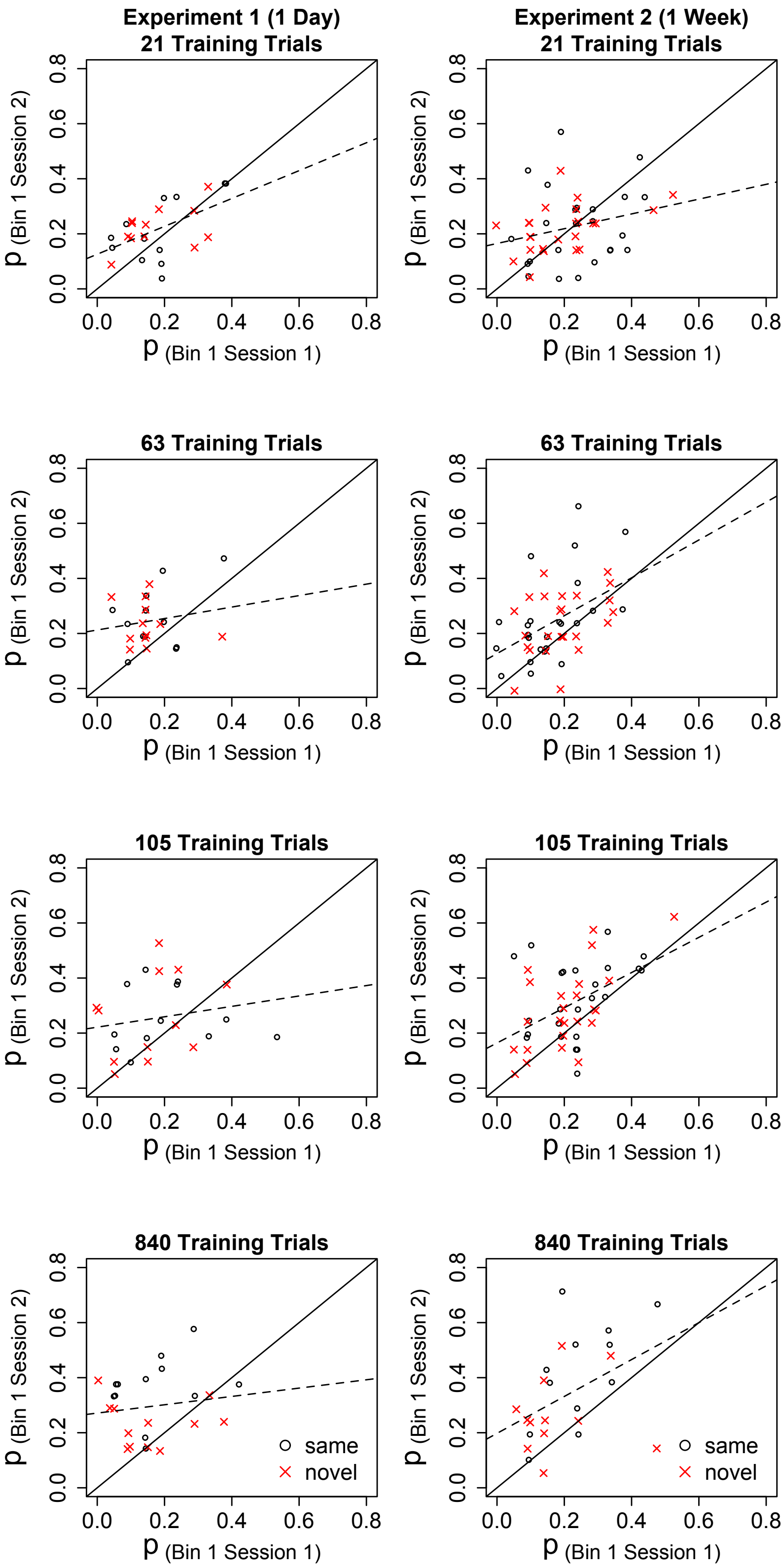

Figure S2: Accuracy in bin 1 of Session 2 plotted against accuracy in bin 1 of Session 1. Each point shows data from a one observer. The dotted line in each plot indicates the linear regression line.
